# Systematic Review of Ticks and Tick-Borne Pathogens of Small Ruminants in Pakistan

**DOI:** 10.3390/pathogens9110937

**Published:** 2020-11-11

**Authors:** Abdul Ghafar, Tariq Abbas, Abdul Rehman, Zia-ud-Din Sandhu, Alejandro Cabezas-Cruz, Abdul Jabbar

**Affiliations:** 1 Department of Veterinary Biosciences, Melbourne Veterinary School, Faculty of Veterinary and Agricultural Sciences, The University of Melbourne, Werribee 3030, VIC, Australia; abdul.ghafar@unimelb.edu.au; 2 Department of Epidemiology and Public Health, Cholistan University of Veterinary and Animal Sciences, Bahawalpur 63100, Punjab, Pakistan; tariqabbas@cuvas.edu.pk; 3 Department of Epidemiology and Public Health, University of Veterinary and Animal Sciences, Lahore 54600, Punjab, Pakistan; abdul.rehman@uvas.edu.pk; 4 Department of Parasitology, University of Agriculture Faisalabad, Faisalabad 38000, Punjab, Pakistan; ziasandhu@hotmail.com; 5 UMR BIPAR, INRAE, ANSES, Ecole Nationale Vétérinaire d’Alfort, Université Paris-Est, 94700 Maisons-Alfort, France; alejandro.cabezas@vet-alfort.fr

**Keywords:** ticks, tick-borne diseases, sheep, goat, anaplasmosis, babesiosis, theileriosis, Q fever, CCHF, Pakistan

## Abstract

Ticks and tick-borne diseases (TTBDis) are a major constraint to the health and production of small ruminants in Pakistan. Despite being the subject of intermittent studies over the past few decades, comprehensive information on the epidemiology and control of TTBDis is lacking. Herein, we have systematically reviewed the current knowledge on TTBDis of small ruminants in Pakistan. Critical appraisal of the selected 71 articles published between 1947 to 2020 revealed that morphological examination had been the most widely used method for the identification of TTBDis in Pakistan. Tick fauna comprise at least 40 species, mainly belonging to *Haemaphysalis*, *Hyalomma* and *Rhipicephalus*. The prevalence of ticks is the highest in summer (June–September) and it is also higher in goats than sheep. *Anaplasma*, *Babesia* and *Theileria* spp. are the major tick-borne pathogens (TBPs), and their prevalence is usually higher in sheep than goats. Spatio-temporal distribution, genetic diversity and control of ticks and TBPs of small ruminants as well as the competence of tick vectors for various TBPs remain to be explored. Therefore, coordinated and focused investigations are required to fill knowledge gaps in these areas to maximise the health, production and welfare of small ruminants and minimise economic losses associated with TTBDis in Pakistan.

## 1. Introduction

Food security is one of the challenges faced by the rapidly growing human population worldwide, particularly in developing countries [1]. For example, the livestock sector plays a crucial role in the national economies and household food security of both developed and developing countries [2], and increasing the production of livestock species (e.g., cattle, sheep and goats) could be one of the ways to address the food shortage in the near future [3]. For this purpose, small ruminants (goat—*Capra hircus* and sheep—*Ovis aries*) are promising livestock species due to their resistance to drought and climatic extremes, low-input production, multipurpose use (for milk, meat and wool) and their ability to utilise household by-products and residues efficiently [4,5]. Small ruminants constitute a major component of the economic, environmental and agricultural niche in various regions of the world, particularly in South Asia, and are an important source of food, livelihood, soil productivity and household stability [6,7].

Among livestock-rearing countries in Asia, Pakistan has the third-largest population of sheep and goats [7], and the national flocks comprise about 28 and 34 breeds of sheep (31.2 million) and goats (78.2 m), respectively, with four main production systems (i.e., nomadic, transhumant, household and sedentary) [8,9,10]. The majority of the small ruminant population is present in the Punjab province (32.6%) followed by Baluchistan (30.6%), Sindh (20.6%) and Khyber Pakhtunkhwa (16.1%) [11], and most of the subsistence landless farmers rear small ruminants as their primary source of income [12]. As sheep and goats are well adapted to diverse climatic and socioeconomic conditions in Pakistan [12], they contribute significantly to the national economy [8]. For example, in the financial year of 2019–2020, 1 million, 0.75 m, 0.47 m and 0.29 m tonnes of milk, meat, wool and hair, respectively, as well as 59.5 m skins of small ruminants, were added to the gross domestic products of Pakistan [8].

Environmental conditions pose various health and production constraints to the optimal production of small ruminants in different climatic zones, i.e., tropical versus temperate climatic zones [13]. For example, in tropics and subtropics, ticks and tick-borne diseases (TTBDis) such as anaplasmosis, babesiosis, ehrlichiosis and theileriosis constitute one of the major health challenges for the production of sheep and goats [14,15,16,17]. Until a decade ago, TTBDis of small ruminants received little attention as most such studies were focused on bovines, possibly due to their higher economic value [18]. However, owing to the recent growing appreciation of the socioeconomic significance of small ruminants in food security and poverty alleviation in resource-poor farming communities globally, more attention is now being directed to the better understanding of TTBDis of sheep and goats [18]. To date, several studies have reported the prevalence of ticks (*Hyalomma*, *Rhipicephalus* and *Haemaphysalis* spp.) [19,20,21,22,23,24,25,26] and tick-borne pathogens (TBPs) (*Anaplasma*, *Babesia* and *Theileria* spp.) in small ruminants from various parts of Pakistan [27,28,29,30,31]. Recently, using a high-throughput microfluidic technique, a broad spectrum of microorganisms in ticks collected from sheep and goats in the Federally Administered Tribal Areas of Pakistan were reported [22]. Moreover, tick-borne zoonotic diseases such as Crimean-Congo haemorrhagic fever (CCHF) and Q fever have also been reported from different parts of Pakistan [32,33,34,35].

Despite the high prevalence and socioeconomic impact of TTBDis of small ruminants in Pakistan, limited information is available about their epidemiology, spatio-temporal distribution and genetic variation, and control measures. Studies on the genetic characterisation of ticks and tick-borne pathogens (TTBPs) are scarce, and more importantly, no information is available on TTBDis from several regions of the country where sheep and goats play a key role in the food security and livelihood of resource-poor farmers. Moreover, no systematic review of the current state-of-the-play of TTBDis of sheep and goats is available from Pakistan. Such an investigation would provide insights into the existing information and help in identifying knowledge gaps and future directions for researchers, and veterinary and medical authorities for the control and prevention of TTBDis. Therefore, this systematic review aims to (i) provide an overview of the existing knowledge on the epidemiology, diagnosis and control of TTBDis and (ii) identify gaps and highlight the future research directions in order to enhance our understanding and control of TTBDis in small ruminants in Pakistan.

## 2. Methods

### 2.1. Review Protocol

The systematic review was conducted following the Preferred Reporting Items for Systematic Reviews and Meta-Analyses (PRISMA) guidelines [36]. Various steps included were the literature search rational, predefined criteria for inclusion and exclusion of the relevance of the references and the extraction of relevant data to achieve the study objectives.

### 2.2. Literature Search

A literature search was conducted for studies published from 1947 to October 2020 on TTBDis of small ruminants in Pakistan using four databases (i.e., Google Scholar, Web of Science, PubMed and CAB Direct). The keywords used for search included tick(s), tick-borne disease(s), tick borne disease(s), small ruminant(s), livestock, sheep, goat(s), haemoparasite(s), anaplasmosis, *Anaplasma*, babesiosis, *Babesia*, theileriosis, *Theileria*, Crimean-Congo haemorrhagic fever, CCHF, Q fever, *Coxiella burnetii*, coxiellosis, and Pakistan. Combinations of various keywords were used to retrieve full-text research articles, postgraduate theses and conference proceedings that reported TTBDis of sheep and goats in Pakistan. Reference lists of retrieved articles were also screened to identify relevant articles (accessed until 14 October 2020).

### 2.3. Quality Assessment and Selection

**Figure 1:**
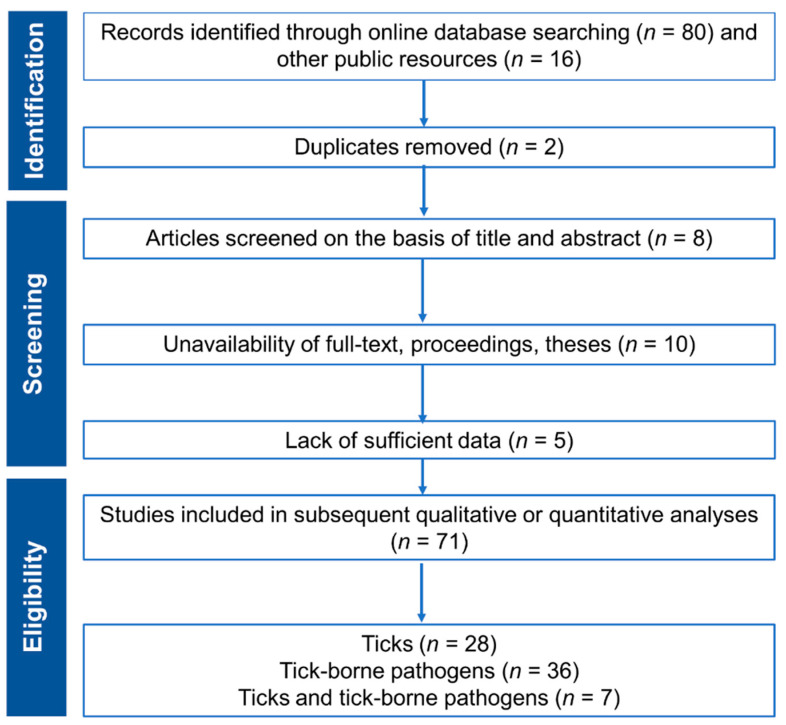
An overview of the Preferred Reporting Items for Systematic Reviews and Meta-Analyses (PRISMA) guidelines for the assessment of peer-reviewed literature and the selection criteria used to select articles for this review paper.

The literature assessment and selection criteria are illustrated in Figure 1. Following the initial identification of references searched through online databases, primary screening was performed based on the titles and abstracts to remove duplicates and irrelevant articles. Full-text articles and theses unavailable online were retrieved through inter-library loans available via the University of Melbourne as well as contacting local libraries in Pakistan. Furthermore, an additional screening step was performed to exclude those articles, theses and conference proceedings that were unavailable as full-text. Where both articles and theses were available, preference was given to published articles. A total of 96 articles related to TTBDis of small ruminants from Pakistan was finally included in this review. However, 25 studies were excluded due to the duplication or poor-quality study design and/or data. Out of 71 eligible studies, 28, 36 and 7 studies were on ticks, TBPs and TTBPs, respectively (Figure 1). Subsequently, data were extracted about the location, study type, study period, host species, tick/pathogen species and reported prevalence. Moreover, attempts were made to extract information about risk factors and interventions, where possible.

### 2.4. Estimation of Prevalence

Prevalence was estimated as the number of hosts infested/infected with at least one individual of a particular parasite divided by the total number of hosts examined for the parasite [37]. We collated the prevalence data on TTBDis from all studies where possible, then estimated the overall prevalence and 95% confidence interval (CI) of TTBPs in different hosts and locations using cumulative population data in Excel spreadsheet (Microsoft 365^®^) and R using the package “binom” [38] following the Clopper–Pearson interval method [39].

## 3. Results and Discussion

### 3.1. Studies on Ticks

**Figure 2:**
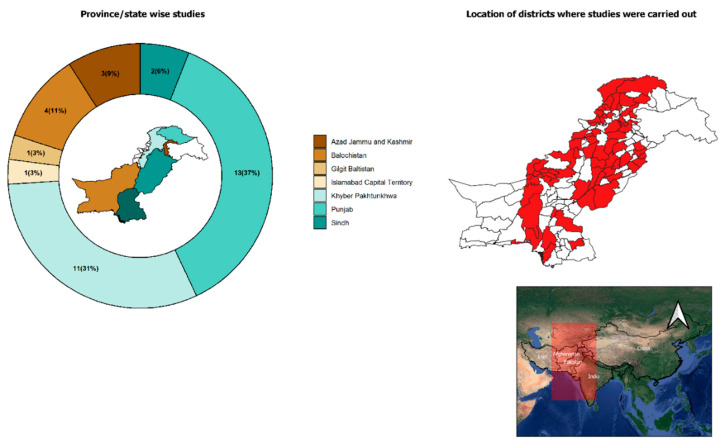
Map of Pakistan (right side) showing the localities where ticks of small ruminants were reported. Donut chart (left side) illustrates the number (percentage in parenthesis) of studies conducted in different provinces, states and the capital territory. Map inside the donut chart indicates boundaries of provinces and states. Inset map shows the location of Pakistan in South Asia.

To date, 28 studies have investigated ticks of sheep and goats in Pakistan whereas only one and six studies focused separately on ticks of goats and sheep, respectively. Details of tick species, host, estimated prevalence, region and identification methods are given in Table 1 and Figure 2.

#### 3.1.1. Tick Species

A total of 40 species belonging to six ixodid (*Amblyomma, Dermacentor, Ixodes, Hyalomma, Haemaphysalis* and *Rhipicephalus*) and three argasid (*Argas*, *Otobius* and *Ornithodoros*) ticks have been reported in sheep and goats from Pakistan (Table 2). The majority of tick species belonged to three genera, i.e., *Hyalomma* (13 species)*, Haemaphysalis* (11 species) and *Rhipicephalus* (8 species) (Table 2).

Data analyses of the reported prevalence estimates [number of studies (*n*) = 21] of ticks in sheep and goats revealed that 27.85% (8032/28,840; range: 0–86.5%; 95% confidence interval (CI): 27.3–28.4%) of the studied population of small ruminants were infested. Tick infestation was apparently higher in goats (30.67%; 5592/18,229; range: 6.7–86.5%; 95% CI: 30.0–31.3%) than sheep (23%; 2440/10,611; range: 0–81.5%; 95% CI: 22.2–23.8%). However, we could not reliably compare the prevalence of ticks in sheep and goats due to the differences in various parameters (such as climate, sample size and target population) of various studies from Pakistan. Although not supported through scientific evidence, sheep wool could provide a barrier against tick infestation on some parts of the body [19,24] whereas agile, restless and grooming characteristics of goat behaviour can make them relatively resistant to ectoparasites such as ticks [40]. Therefore, both sheep and goats could be equally susceptible to tick infestation in the subtropical conditions of Pakistan.

#### 3.1.2. Epidemiology of Ticks

To date, the majority of studies (24/35) aimed at the epidemiology of ticks have been conducted in Punjab and Khyber Pakhtunkhwa, whereas only 1–4 studies were available from other regions which inhabit more than half of the population of small ruminants in Pakistan [11]. To the best of our knowledge, the first detailed account on the morphological characterisation of ticks in various mammalian hosts (including livestock, companion and wild animals) from Pakistan was provided by McCarthy [41]. However, this study did not provide information on the prevalence and burden of ticks from various host species. Subsequently, the majority of studies conducted adopted a convenience or opportunistic sampling strategy and/or covered smaller geographical (mostly peri-urban or near veterinary institutions in major metropolitan areas) zones targeting smaller sample sizes to investigate ticks in small ruminants [19,20,21,22,23,24,25,26,27,42,43,44,45,46,47,48,49,50,51,52,53,54,55,56,57,58,59,60,61,62,63,64,65,66].

Seasonal variation of tick infestation in a region is dependent upon the fluctuation of monthly/annual temperature and moisture [67]. To date, a few studies have assessed the seasonal variation in tick prevalence in small ruminants in Pakistan and reported a higher tick infestation in summer (June–September) [23,25,50,51,53,56,58,64,65,66], possibly due to higher temperature- and moisture-levels suitable for the development of ticks [68,69]. A number of risk factors can favour the tick infestation in animals [19,70] and only two studies have investigated risk factors associated with tick infestation of sheep and goats in Pakistan [19,53]. These authors found that traditional housing, free grazing, the lack of acaricidal drug use and the absence of rural poultry were the main risk factors for the higher occurrence of ticks in small ruminants [19,53]. Furthermore, tick prevalence is affected by climatic conditions across different agro-ecological zones (AEZs) of a region/country [71]. Recently, we demonstrated a significant variation in the prevalence (22.2–70.5%; *p* < 0.0001) of bovine ticks (*Hy. anatolicum*, *Hy. hussaini*, *Hy. scupense*, *Rh. annulatus* and *Rh. microplus*) across five AEZs of Pakistan [72]. However, for small ruminants, only a small population of sheep [number of individuals (*N* = 18)] and goats (*N* = 80) from arid and semi-arid AEZs of Punjab province [19] was examined for tick infestation. Given that the tick infestation of animals can be influenced by various factors such host, husbandry, management and environment, it is pivotal to enhance our understanding of the epidemiology of ticks of small ruminants by assessing agro-climatic and spatio-temporal differences across various AEZs of Pakistan so that effective and sustainable control programs for TTBDis of small ruminants could be developed.

#### 3.1.3. Identification of Ticks

Accurate identification of ticks is central to the understanding of the epidemiology of TTBDis and developing effective control strategies [73]. Morphological characterisation using dichotomous keys has been the most commonly used method to identify ticks to species level (22/25) followed by a combination of microscopic and molecular methods (3/25) (Table 1). However, six studies provided only genus-level identification of ticks [27,46,49,54,57,65] whereas three studies did not provide information on the identification of ticks [43,45,64]. Although the microscopic examination of ticks is simple and cheap, it has several limitations such as the requirement of entomological expertise for the identification of closely-related species (e.g., *Rh. sanguineus* and *Rh. turanicus*; *Hy. anatolicum* and *Hy. excavatum*), immature or larval stages, and engorged or damaged specimens [74,75,76,77]. Such limitations could sometimes lead to unreliable data, including reports of non-endemic tick species such as those of *Rh. appendiculatus* [42] and *Amblyomma hebraeum* [58] from Pakistan. Molecular characterisation and analyses of short regions of genetic material (known as DNA barcode) can provide an alternative approach to species-level identification [76,78]. For this purpose, several nuclear (second internal transcribed spacer) and mitochondrial (cytochrome *c* oxidase subunit I, 12S and 16S ribosomal RNA) markers have been utilised successfully worldwide [22,79,80,81,82]. During the last decade, matrix-assisted laser desorption/ionisation time-of-flight mass spectrometry (MALDI-TOF MS) has also been successfully used for the identification of ticks [73,83]. However, due to the limitations associated with each method, no single method is ideal for accurate characterisation of ticks. Therefore, the use of a combination of morphological and molecular methods would be essential for the surveillance and control of ticks of veterinary and public health significance in Pakistan.

#### 3.1.4. Control of Ticks in Small Ruminants

There is a scarcity of data on method(s) used for the control of ticks in small ruminants from Pakistan, partly because farmers pay less attention to the husbandry and management of sheep and goats than those of bovines due to the higher economic value of latter (Ghafar et al. unpublished data). Despite the serious environmental and health implications associated with acaricides (such as macrocyclic lactones, trichlorfon and cypermethrin), their periodic application is the main tick control method used in small and large ruminants in Pakistan [14,84,85]. Additionally, grooming, i.e., manual picking of ticks by the farm workers, is also commonly practised for tick control in Pakistan [19]. To date, only two studies have assessed the in vivo efficacy of acaricidal drugs, including coumaphos, cypermethrin, diazinon and ivermectin [48,53], and these authors concluded that cypermethrin was the most effective drug against ticks in both sheep and goats (Table 3). 

**Table 1:** List of key studies of ticks of small ruminants in Pakistan.

State(s)	District(s)	Host(s)	Tick(s)	Method(s) of Identification	% Infested Animals (Proportion; 95% Confidence Interval)	Reference
Khyber Pakhtunkhwa	Charsadda, Karak, Mardan, Lower Kohistan, Peshawar	Sheep	*Haemaphysalis longicornis, Hyalomma impeltatum*	Morphological	16.3 (13/80; 8.2–24.3)	[23]
		Goats	*Hae. montgomeryi, Hae. longicornis,* *Hy. impeltatum*		68.3 (82/120; 60.0–76.7)	
	Peshawar	Sheep	*Dermacentor variabilis, Ixodes ricinus, Rhipicephalus simus, Otobius megnini*	Molecular	Not provided	[42]
		Goats	*Rh. appendiculatus, Rh. microplus,* *Rh. simus*			
	Bannu	Sheep	Not provided	Not performed	7.8 (39/500; 5.4–10.2)	[43]
		Goats			10.2 (51/500; 7.5–12.9)	
	Bajaur, Khyber, Mohmand, Orakzai, North and South Waziristan	Sheep, Goats	*Hae. sulcata, Hae. punctata,* *Hy. anatolicum, Hy. detritum,* *Hy. excavatum, Hy. scupense,* *Rh. microplus, Rh. sanguineus*	Morphological	Not Provided	[21]
	Karak	Sheep	*Hy. marginatum, Rh. annulatus*	Morphological	26.7 (8/30; 10.8–42.5)	[44]
		Goats	*Hae. bispinosa, Rh. microplus,* *Rh. sanguineus*		20.0 (9/45; 8.3–31.7)	
	Dera Ismail Khan, Lakki Marwat	Sheep	Not provided	Not performed	27.3 (9/33; 12.1–42.5)	[45]
		Goats	Not provided		23.1 (34/147; 16.3-29.9)	
	Peshawar	Sheep	*Amblyomma, Boophilus, Haemaphysalis, Ixodes and Rhipicephalus* species	Morphological	66.7 (50/75; 56.0–77.3)	[46]
		Goats			73.7 (70/95; 64.8–82.5)	
	Bannu, Chitral, Dir, Mardan, Peshawar, Swat	Sheep	*D. raskemensis, Hy. anatolicum,* *Hy. detritum, Rh. microplus, Rh. sanguineus*	Morphological	Not provided	[47]
		Goats	*Hae. montgomeryi, Hy. anatolicum,* *Hy. marginatum turanicum,* *Rh. haemaphysaloides, Rh. microplus, Rh. sanguineus*			
	Mansehra	Sheep	*Rh. sanguineus*	Morphological	Not provided	[48]
		Goats				
	Bajaur, Khyber, Mohmand, North Waziristan, Orakzai	Sheep	*Hae. sulcata, Hy. anatolicum,* *Rh. microplus, Rh. turanicus*	Morphological and molecular	Not provided	[22]
		Goats	*Hae. punctata, Hae. sulcata, Hy. anatolicum, Rh. haemaphysaloides,* *Rh. microplus*, *Rh. turanicus*			
Khyber Pakhtunkhwa and Gilgit-Baltistan	Astor, Diamer, Gilgit, Haripur, Kohistan, Mansehra, Shangala	Sheep	*Hyalomma* and *Rhipicephalus* spp.	Morphological	81.5 (189/232; 76.5–86.5)	[49]
		Goats			72.1 (263/365; 67.5–76.7)	
Punjab	Attock, Bahawalpur, Bhakkar, Chakwal, Faisalabad, Gujranwala, Jhang, Khushab, Layyah, Muzaffargarh, Rajanpur, Rawalpindi	Sheep	*Hy. anatolicum, Rh. appendiculatus,* *Rh. decolaratus, Rh. microplus,* *Rh. sanguineus*	Morphological	29.0 (812/2800; 27.3–30.7)	[25]
		Goats	*Hy. anatolicum, Hy. dromedarii,* *Hy. marginatum, Rh. appendiculatus, Rh. decolaratus, Rh. microplus,* *Rh. sanguineus*		36.1 (1012/2800; 34.4–37.9)	
	Toba Tek Singh	Goats	*Hy. anatolicum, Rh. microplus*	Morphological	6.7 (270/4020; 5.9–7.5)	[50]
	Sargodha	Goats	*Hy. anatolicum* and *Amblyomma, Haemaphysalis, Ixodes,* and *Rhipicephalus* spp.	Morphological	86.5 (1038/1200; 84.6–88.4)	[51]
	Multan	Sheep	*Hy. anatolicum, Hy. marginatum,* *Rh. sanguineus*	Morphological	68.0 (17/25; 49.7–86.3)	[52]
		Goats			40.0 (8/20; 18.5–61.5)	
	Multan	Sheep	*Hae. punctata, Hy. anatolicum,* *Hy. excavatum*	Morphological	50.0 (100/200; 43.1–56.9)	[26]
		Goats	*Hy. excavatum, Rh. microplus*		40.8 (102/250; 34.7–46.9)	
	Attock, Bahawalpur, Gujranwala, Kasur, Khanewal, Multan, Okara, Rahim Yar Khan, Vehari	Sheep	*Hy. anatolicum, Rh. microplus*	Morphological and molecular	11.1 (2/18; 1.3–34.7)	[19]
		Goats	*Hy. anatolicum, Hy. dromedarii,* *Rh. microplus, Rh. turanicus*		60.0 (48/80; 49.3–70.7)	
	Layyah, Muzaffargarh	Sheep	No ticks found	Morphological	0.0 (0/1400; 0.0–0.2)	[24]
		Goats	*Hy. anatolicum, Rh. sanguineus*		51.6 (723/1400; 49.0–54.3)	
	Layyah, Muzaffargarh	Goats	*Hy. anatolicum, Rh. sanguineus*	Morphological	60.1 (481/800; 56.7–63.5)	[53]
	Lahore	Sheep	*Boophilus, Hyalomma* and *Rhipicephalus* spp.	Morphological	Not provided	[27]
	Multan	Goats	*Haemaphysalis* and *Rhipicephalus* spp.	Morphological	43.4 (201/463; 38.9–47.9)	[54]
	Faisalabad, Jhang, Toba Tek Singh	Sheep	*D. marginatus, Hy. anatolicum,* *Hy. marginatum isaaci, Rh. annulatus, Rh. microplus, Rh. sanguineus*	Morphological	18.8 (846/4500; 17.7–19.9)	[55]
		Goats	*Hy. aegyptium, Hy. anatolicum,* *Hy. marginatum isaaci, Rh. annulatus, Rh. microplus, Rh. sanguineus*		12.3 (553/4500; 11.3–13.2)	
	Lahore, Sheikhupura	Sheep	*Hae. burnati, Hy. anatolicum,* *Rh. annulatus, Rh. microplus,* *Rh. sanguineus*	Morphological	Not provided	[56]
		Goats				
Punjab and Islamabad Capital Territory	Livestock experimental stations located in Attock and Islamabad Capital Territory	Sheep	*Haemaphysalis* and *Rhipicephalus* spp.	Morphological	43.4 (95/219; 36.8–49.9)	[57]
		Goats	*Amblyomma, Haemaphysalis, Ixodes* and *Rhipicephalus* spp.		41.5 (184/443; 36.9–46.1)	
Balochistan	Harnai	Sheep	*Hy. anatolicum, Hy. dromedarii,* *Rh. annulatus, Rh. microplus*	Morphological	30.0 (12/40; 15.8–44.2)	[58]
		Goats	*Am. hebraeum, Hy. anatolicum,* *Hy. dromedarii, Rh. annulatus*		27.5 (11/40; 13.7–41.3)	
	Mustang and Quetta	Goats	*Hy. anatolicum, Hy. excavatum,* *Rh. appendiculatus, Rh. microplus*	Morphological	Not provided	[59]
	Specimens collected from 26 districts (names not provided)	Sheep and Goats	*Hae. flava, Hy. anatolicum*	Morphological	Not provided	[60]
	Harnai, Kalat, Killa Abdullah, Khuzdar, Lasbela, Loralai, Pishin, Quetta, Sherani, Sibi, Ziarat, Zhob	Sheep	*Hy. anatolicum, Hy. dromedarii,* *Hy. excavatum, Hy. marginatum,* *Hy. scupense, Rh. microplus,* *Rh. turanicus*	Morphological and molecular	Not provided	[61]
		Goats	*Hy. anatolicum, Hy. dromedarii,* *Hy. excavatum, Hy. marginatum*			
Sindh	Khairpur, Larkana, Sehwan, Thatta, Umerkot	Sheep	*Hae intermedia, Hae kutchensis,* *Hae. bispinosa, Hy. anatolicum,* *Hy. bravepunctata, Hy. detritum,* *Hy. dromedarii, Hy. hussaini,* *Hy. impeltatum, Hy. marginatum isaaci, Hy. marginatum turanicum,* *Rh. annulatus, Rh. haemaphysaloides, Rh. microplus, Rh. sanguineus,* *Rh. turanicus*	Morphological	Not provided	[62]
		Goats				
	Khairpur	Goats	*Hy. anatolicum, Hy. dromedarii,* *Hy. impeltatum, Hy. marginatum isaaci, Rh. haemaphysaloides, Rh. turanicus*	Morphological	Not provided	[63]
Azad Jammu and Kashmir	Muzaffarabad	Sheep	Not provided	Not performed	22.2 (2/9; 2.8–60.0)	[64]
		Goats			46.3 (19/41; 31.1–61.6)	
	Poonch	Sheep	*Haemaphysalis, Hyalomma* and *Otobius* spp.	Morphological	54.7 (82/150; 46.7–62.6)	[65]
		Goats			48.3 (145/300; 42.7–54.0)	
	Poonch	Sheep	*Hy. anatolicum*	Morphological	54.7 (164/300; 49.0–60.3)	[66]
		Goats			48.0 (288/600; 44.0–52.0)	
Azad Jammu and Kashmir, Balochistan, Gilgit Baltistan, Khyber Pakhtunkhwa, Punjab and Sindh	District information not provided	Sheep	*Ar. persicus, Hae. bispinosa,* *Hae. cornupunctata, Hae kashmirensis, Hae. montomeryi, Hy. anatolicum,* *Hy. dromedarii, Hy. hussaini, Hy. isaaci, Hy. scupense, Or. tholozani,* *Rh. haemaphysaloides, Rh. microplus*	Morphological	Not provided	[20]
		Goats	Same as above except *Argas* (*Ar*.) *persicus* absent			
Azad Kashmir, Balochistan, Gilgit Baltistan, Khyber Pakhtunkhwa, Punjab and Sindh	Specimens were collected from 12 administrative divisions of West Pakistan and Azad Kashmir	Sheep	*D. raskemensis, Hae. bispinosa,* *Hae. cornupunctata, Hae. kashmirensis, Hae. montgomeryi, Hae. sulcata,* *Hy. anatolicum, Hy. asiaticum,* *Hy. detritum, Hy. dromedarii,* *Hy. excavatum*, *Hy. kumari, Hy. marginatum isaaci, Hy. marginatum turanicum, Rh. annulatus, Rh. haemaphysaloides, Rh. microplus,* *Rh. sanguineus, Rh. turanicus*	Morphological	Not provided	[41]
		Goats	Same species as above except *Hy. excavatum* and *Rh. annulatus* absent			

**Table 2:** Ticks and tick-borne pathogens (TBPs) of small ruminants in Pakistan.

Ticks		Number of Species Reported	Selected References
*Hyalomma* (*Hy.*)	*Hy. anatolicum, Hy. asiaticum, Hy. bravepunctata, Hy. detritum, Hy. dromedarii, Hy. excavatum, Hy. hussaini, Hy. impeltatum, Hy. kumari, Hy. marginatum, Hy. marginatum isaaci, Hy. marginatum turanicum, Hy. scupense*	13	[20,21,22,23,25,41,47,55]
*Rhipicephalus* (*Rh.*)	*Rh. annulatus, Rh. appendiculatus, Rh. decolaratus, Rh. haemaphysaloides, Rh. microplus, Rh. sanguineus, Rh. simus, Rh. turanicus*	8	[20,21,22,25,41,47,55]
*Haemaphysalis* (*Hae.*)	*Hae. burnati, Hae. bispinosa, Hae. cornupunctata, Hae. flava, Hae. intermedia, Hae. kashmirensis, Hae. kutchensis, Hae. longicornis, Hae. montgomeryi, Hae. punctata, Hae. sulcata*	11	[20,21,22,23,41,47]
Other ixodids and argasids	*Amblyomma hebraeum, Dermacentor marginatus, D. variabilis, D. raskimensis, Ixodes ricinus, Ar*. *persicus, Otobius megnini, Ornithodoros tholozani*	8	[20,41,47,55]
**Tick-Borne Pathogens**			
*Anaplasma* (*A.*)	*A. centrale, A. marginale, A. ovis*	3	[22,86,87]
*Babesia* (*B*.)	*B. ovis*	1	[28,88]
*Theileria* (*T*.)	*T. annulata, T. luwenshuni, T. ovis, T. lestoquardi, T.* sp. *MK, T.* sp. *OT1*	6	[27,89]
*Rickettsia* (*R.*)	*Candidatus R. amblyommii, R. aeschlimannii, R. conorii, R. massiliae, R. slovaca,*	5	[20,22]
Other pathogens	*Coxiella burnetii*, Crimean-Congo haemorrhagic fever virus	2	[32,34,35]

**Table 3:** Drug efficacy trials against ticks and tick-borne diseases of small ruminants in Pakistan.

Study Type	Drug(s) Tested (Concentration/Dose/Method of Application)	Number of Animals per Group		Duration of Trial (Days)	Efficacy (%)		Reference
		Sheep	Goats		Sheep	Goats	
Acaricidal efficacy against ticks	Diazinon (0.6% spray)	20	20	56	89.5	92	[48]
	Coumaphos (0.1% spray)	20	20		93.6	95	
	Cypermethrin (2% spray)	20	20		100	100	
	Ivermectin (0.2 mg/kg, injection)	NS	90	20	NA	No	[53]
	Cypermethrin (5% spray)		90			Yes	
Drug efficacy against anaplasmosis	Oxytetracycline (1 mL/kg, injection)	NS	10	30 *	NA	30	[98]
	Imidocarb dipropionate (0.1/kg, injection)		10			80	
	Diminazene aceturate (0.3 mL/kg, injection)		10			60	
	Oxytetracycline (20 mg/kg, injection)	4	4	10	100	100	[87]
	Imidocarb dipropionate (3 mg/kg, injection)	4	4		100	87.5	
	Diminazene aceturate (3.5–7 mg/kg, injection)	4	4		50	75	
Drug efficacy against babesiosis	Imidocarb dipropionate + oxytetracycline (2 mg/kg + 10 mg/kg, injection)	10	10	10	100	100	[99]
	Imidocarb dipropionate (2 mg/kg, injection)	10	10		80	80	
	Diminazene aceturate + oxytetracycline (3.5 mg/kg + 10 mg/kg, injection)	10	10		80	90	
	Diminazene aceturate (3.5 mg/kg, injection)	10	10		70	70	
	Imidocarb dipropionate (2 mg/kg, injection)	10	NS	10	100	NA	[100]
	Diminazene aceturate (3.5 mg/kg, injection)	10			80		

* Three doses of each drug were given in this study whereas a single dose was administered in the rest of listed studies; NS; Not studied; NA: Not applicable.

Owing to the limited understanding of spatio-temporal epidemiology of ticks of small ruminants in Pakistan, no regular tick control program is followed [14]. During the last decade, due to increasing cases of CCHF infections in humans, event-based tick control campaigns (known as anti-Congo campaign) were launched every year by the provincial governments just before Eid-ul-Adha—a religious festival of Muslims when they slaughter animals at their homes [90,91,92]. Additionally, sporadic campaigns are also common during the summer season. These campaigns involved the repetitive use of same acaricidal drugs (mostly injectable ivermectin and/or cypermethrin spray) over the years which could possibly have contributed to the development of acaricidal resistance in ticks as reported from elsewhere [53,93,94,95]. There is a need to test the efficacy of alternative acaricidal drugs and other prophylactic measures such as tick vaccines [96]. Furthermore, future studies investigating the status of acaricidal resistance in tick populations of small ruminants would guide integrated control of ticks in this country. Such integrated tick control strategies, consisting in the systematic combination of at least two control technologies, including anti-tick recombinant vaccines, aiming to reduce selection pressure in favour of acaricide-resistant individuals, while maintaining adequate levels of animal production, have been implemented in some countries such as Cuba with promising results [97].

### 3.2. Tick-Borne Pathogens in Pakistani Small Ruminants and Their Ticks

**Figure 3:**
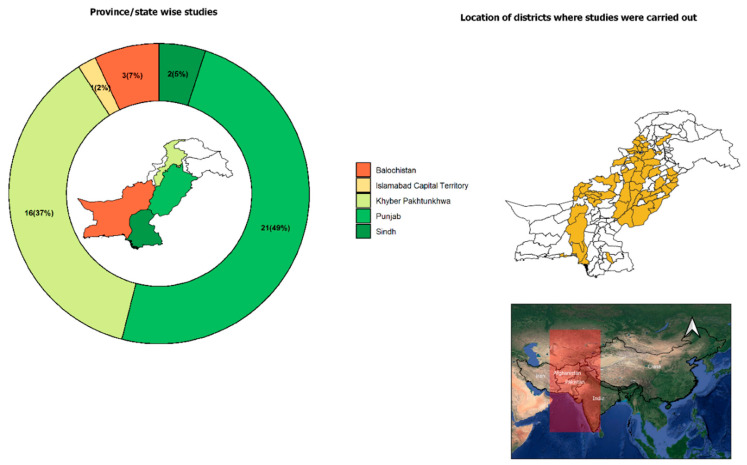
Map of Pakistan (right side) showing localities from where TBPs of small ruminants were reported. Donut chart (left side) illustrates the number (percentage in parenthesis) of studies conducted in different provinces, states and the capital. Map inside the Donut chart indicates boundaries of provinces and states. Inset map shows the location of Pakistan in South Asia.

To date, bacterial (anaplasmosis and Q fever) protozoal (babesiosis and theileriosis) and viral (CCHF) TBDs of veterinary and public health significance have been reported in small ruminants as well as their ticks from Pakistan (Figure 3, Table 2, Table 4, Table 5 and Table 6). The following sections provide an overview of the key TBDs of ruminants in Pakistan.

#### 3.2.1. Anaplasmosis

Anaplasmosis is one of the most important TBDs of livestock in Pakistan [14] and, in small ruminants, it is caused by members of an intracellular, Gram-negative bacteria, *Anaplasma* (Rickettsiales: Anaplasmataceae; *A. ovis*, *A. phagocytophilum* and *A. marginale*) [101,102]. It is transmitted by various genera of ticks, including *Dermacentor*, *Hyalomma*, *Haemaphysalis* and *Rhipicephalus* [103,104,105]. Infections with *A. ovis* in sheep and goats are characterised by haemolytic anaemia and a low-grade fever, respectively [96]. In Pakistan, only two studies have reported the detection of *A. ovis* DNA (using microfluidic real-time polymerase chain reaction (PCR) and reverse line blot assay) in *Rhipicephalus*, *Hyalomma* and *Haemaphysalis* ticks of small ruminants [22,86]. However, there is no experimental evidence for the transmission of *A. ovis* by these or any other tick species from Pakistan.

To date, a total of 12 studies (Punjab = 3; Khyber Pakhtunkhwa = 8; Sindh = 1) has reported the occurrence of three *Anaplasma* species (*A. ovis*, *A. marginale* and *A. centrale*) in small ruminants (*n* = 10) [29,87,98,106,107,108,109,110,111,112] and ticks (*n* = 2) [22,86] from Pakistan (see Table 4). Based on these studies, the estimated overall prevalence of anaplasmosis in Pakistani small ruminants are 1.7–55.3% and 25.3–47.2% using microscopic and molecular methods, respectively. However, slightly higher prevalences were reported in sheep (13.9–55.3%; 23.9–36.8%; and 28–47.2%) than goats (1.7–30.7%; 20.6–32.8%; and 25.3–34.8%) using microscopic, serological (enzyme-linked immunosorbent assay [ELISA]) and molecular methods, respectively. This higher prevalence in sheep could possibly be due to a higher susceptibility of sheep to clinical anaplasmosis than goats [105]. The prevalence of *Anaplasma* spp. in both sheep and goats was the highest (25.3–55.3%) in Punjab province followed by Khyber Pakhtunkhwa (1.7–47.2%) and Sindh (13.3%). To date, only one study has estimated the occurrence of *Anaplasma* spp. in ticks of small ruminants from Khyber Pakhtunkhwa province which reported a higher occurrence of the bacteria in ticks from sheep (39.1%) than those collected from goats (35.5%) [22]. Despite a widespread occurrence and the reported higher prevalence of *A. ovis*, the epidemiology of anaplasmosis in Pakistani small ruminants is poorly-understood, probably due to mild and/or asymptomatic infections in sheep and goats as well as the lack of record keeping by small-holder farmers [113,114]. Given the recently identified zoonotic potential of *A. ovis* and its ability to cause severe clinical disease particularly when present as a co-infection [113,115], future research should focus on understanding the disease epidemiology and vector competence of potential ticks known to infest small ruminants in different AEZs of the country.

#### 3.2.2. Babesiosis

Babesiosis, caused by intraerythrocytic protozoa of the genus *Babesia* (Piroplasmida: Babesiidae), is one of the most common and economically important TBD of domestic and wild ruminants worldwide [116]. The disease is mainly transmitted by *Dermacentor*, *Hyalomma*, *Haemaphysalis* and *Rhipicephalus* ticks [17,105]. In sheep and goats, clinical babesiosis is caused by *Babesia ovis*, *B. motasi* and yet unidentified *Babesia* sp. (China) whereas *B. crassa* is usually associated with mild infections [105]. Acute babesiosis is characterised by high fever, anaemia, tachycardia, jaundice, haemoglobinuria, abdominal pain and death [104]. Higher infection rates and severe clinical manifestations are more common in sheep than goats [17,105,117]. To date, only *B. ovis* has been reported in small ruminants in Pakistan [28,88,118]. Similarly, *B. ovis* has been detected using the microscopic examination of the haemolymph of *Rh. sanguineus* collected from sheep and goats [48] as well as from a tick collected from bovines, *Hy. anatolicum* using a microfluidic-based real-time PCR [119].

Among all major TBDs of small ruminants in Pakistan, babesiosis is the least-studied disease (Punjab = 5; Khyber Pakhtunkhwa = 4) (see Table 5). Based on the available data on the occurrence of babesiosis in small ruminants, 7–41.7% and 23.9–55% of the studied population of goats and sheep was positive using microscopic and molecular methods, respectively. The prevalence of babesiosis was variable in sheep (7–29% and 50–55%) and goats (13.5–41.7% and 23.9%) using microscopic and molecular methods, respectively. Like anaplasmosis, the higher prevalence of babesiosis (using molecular methods) in sheep could be due to the natural resistance of goats to TBDs [17,105,120]. To date, only one study has reported the occurrence of *B. ovis* in ticks (microscopic examination) from sheep (1.5%) and goats (1%) [48] whereas the molecular screening of a small number of ticks (*N* = 54) from small ruminants in Khyber Pakhtunkhwa did not detect *B. ovis* [22]. Without the large-scale epidemiological investigation of caprine and ovine *Babesia* species using high-throughput techniques, it is not possible to assess the level of risks associated with babesiosis in small ruminants from Pakistan.

#### 3.2.3. Theileriosis

Theileriosis is caused by members of genus *Theileria* (Piroplasmida: Theileridae) and three pathogenic (*Theileria lestoquardi*, *T. luwenshuni* and *T. uilenbergi*) and three non-pathogenic (*T. ovis*, *T. separate* and *T. recondite*) species are known to infect small ruminants [121]. Transmission occurs via tick species belonging to three main genera, *Haemaphysalis, Hyalomma* and *Rhipicephalus* [122]. In most parts of the world, malignant theileriosis in sheep and goats is caused by *T. lestoquardi* and is transmitted by *Hy. anatolicum* [121], and the main clinical signs include anorexia, anaemia, naso-lacrimal discharge, fever, emaciation, enlarged prescapular lymph nodes, haemoglobinuria, cardiac dysfunction and even death [123,124].

Theileriosis is the most studied TBD of small ruminants in Pakistan, with 20 investigations in vertebrate animals [27,28,30,31,54,57,88,89,107,123,125,126,127,128,129,130,131,132,133,134] and three in ticks [22,27,86] from Punjab (*n* = 11), Khyber Pakhtunkhwa (*n* = 10), the Islamabad Capital Territory (*n* = 1) and Balochistan (*n* = 1) (see Table 5). The most frequently reported *Theileria* species are *T. ovis* and *T. lestoquardi*; whereas, a recent study also reported *T. luwenshuni, Theileria* sp. *MK*, and *Theileria* sp. OT1 in sheep and goats [89]. Based on the previous studies, the overall estimated prevalence of theileriosis was 1–22% and 0–72.5% using microscopic and molecular methods, respectively. Like anaplasmosis and babesiosis, the comparatively higher prevalence was reported in sheep (1–22% and 4.5–72.5%) than goats (3.0–8.2% and 0.0–69.1%) using microscopic and molecular, methods, respectively. Contrarily, based on the findings of a single study, the prevalence of *Theileria* species was slightly higher in ticks from goats (35.5%) compared to those from sheep (30.4%) [22]. Despite the higher reported prevalence of ticks in goats and pathogens in ticks collected from goats, lower prevalence of TBDs in goats indicate their natural resistance [17,104,119]. As discussed above, future information on the spatio-temporal epidemiology of theileriosis would be pivotal for the control of TBDs of small ruminants in Pakistan.

#### 3.2.4. Other Tick-Borne Diseases (TBDs) of Small Ruminants

A total of eight studies has investigated other important TBDs, including coxiellosis and CCHF in ticks and small ruminants [20,22,32,33,34,35,61,134] (Table 6). Coxiellosis (Q fever) was reported mainly from Punjab (*n* = 3) and Sindh (*n* = 1), with an overall prevalence of 4.6–33.2% and 7.7–31% in animals (using serological methods, i.e., ELISA and complement fixation test [CFT]) and ticks (using qualitative PCR), respectively [33,34,135,136]. The prevalence of coxiellosis was comparatively higher in sheep (15.6–33.2% and 18.3% using ELISA and CFT, respectively) than goats (15–28.4% and 4.6% using ELISA and CFT, respectively). Similarly, the higher prevalence was reported in ticks collected from sheep (31%) than those from goats (7.7%). In small ruminants, coxiellosis is usually asymptomatic or sub-clinical but sometimes could lead to reproductive disorders, including premature or weak offspring, abortion, and stillbirths [34]. Nonetheless, infected small ruminants are considered as a source of infection for humans [137]. Zoonotic significance of this disease is quite high and the limited data from Pakistan shows a significant prevalence (10.2–26.8%) in the human population [34]. Moreover, a recent study detected *Coxiella burnetii* (1.94%; 47/2425) in soil samples using qPCR from nine districts in Punjab [136].

Another major tick-borne zoonotic disease in Pakistan is CCHF and only a few studies have detected this virus in blood (1%, 8/800) and ticks (3.8%, 20/525) collected from sheep [61], and specific antibodies in sheep (18.6–32.5%) and goats (4.6–18.9%) [32,35]. In Pakistan, the incidence of CCHF is usually higher in urban areas before Eid-ul-Adha when people slaughter animals as a religious ritual [90,92]. Moreover, it is believed that the last two decades of the Afghan war also resulted in a large influx of refugees along with their livestock, leading to an increase in CCHF cases [92]. In small ruminants, CCHF is usually asymptomatic, but it can be life-threatening in humans [138], who usually become infected upon exposure to a vector (*Hyalomma*) or body fluids of the infected animals [138]. In Pakistan, farmers and veterinarians are at a higher risk of CCHF due to the limited knowledge of the disease and its transmission, high tick prevalence—particularly ticks of the genus *Hyalomma* which is the principal vector for CCHFV—on small-scale farms, poor diagnostic facilities and the lack of control and preventive measures for both ticks and the virus [72,92].

#### 3.2.5. Diagnosis and Control of TBPs in Pakistan

Microscopic examination of Giemsa-stained blood smears is considered the gold standard for the diagnosis of haemoparasitic infections worldwide [139]. Several serological assays (such as ELISA, indirect fluorescence assay and CFT) are also available for the detection of antigens or antibodies against TBPs [106,140,141,142,143]. However, microscopic and serological methods are of limited value due to several limitations, including lower sensitivity and specificity, cross-reactivity, inability to detect carrier infections, and the requirement of expertise and time [140,144,145]. These limitations have been overcome through the use of highly sensitive molecular methods, including conventional PCR (cPCR), quantitative PCR (qPCR), nested PCR (nPCR), reverse line blotting (RLB), loop-mediated isothermal amplification (LAMP), high-resolution melting (HRM) assays, high-throughput microfluidics-based real-time PCR and the next-generation sequencing (NGS) [105,146,147,148,149,150].

In Pakistan, microscopy is the most commonly used method for the detection of TBPs in scientific studies. However, field diagnosis is usually made based on clinical signs and the history of tick exposure, mainly due to the unavailability of well-equipped veterinary diagnostic laboratories in the country [14,114,151]. To date, a few studies have used serological (ELISA, indirect fluorescence assay (IFA) and CFT) and molecular methods (cPCR, qPCR, nPCR and microfluidic real-time PCR); however, the sequencing of PCR amplicons has rarely been performed, thereby no detailed information is available on the genetic diversity of TBPs in small ruminants from Pakistan. Moreover, a number of studies also investigated changes in the haematological profiles of animals infected with TBPs and reported a decrease in haemoglobin and packed cell volume associated with anaplasmosis, babesiosis and theileriosis [30,31,87,106,133].

Control of TBDs in small ruminants mainly relies on the use of acaricides (listed under the Section 3.1.4), antibiotics (oxytetracycline) and antiprotozoal drugs (imidocarb dipropionate, diminazene aceturate and buparvaquone) [139,152]. In Pakistan, data on the control of TBDs in small ruminants is scarce and acaricidal drugs are used to control ticks [53], whereas clinical cases of anaplasmosis, babesiosis and theileriosis are usually treated with a combination of babesicidal and theilericidal drugs and/or antibiotics. A few studies have tested the efficacy of various drugs against TBDs of small ruminants in Pakistan and reported that imidocarb dipropionate and oxytetracycline/diminazene aceturate were effective against babesiosis [99,100] and anaplasmosis [53,87], respectively.

**Table 4:** List of key studies of anaplasmosis of small ruminants in Pakistan.

State	District(s)	Host(s)/Vector	Method(s) of Detection	Target Animal Population	Pathogen(s) Detected	% Test-Positive (Proportion; 95% CI)	Reference
Punjab	Lahore	Sheep	Morphological	Suspected of anaplasmosis	*Anaplasma ovis*	55.3 (83/150; 47.4–63.3)	[87]
		Goats				30.7 (46/150; 23.3–38.0)	
	Mianwali	Sheep	Molecular	Healthy	*Anaplasma* species	32.0 (24/75; 21.4–42.6)	[106]
		Goats				25.3 (19/75; 15.5–35.2)	
		Sheep and goats	Morphological			29.3 (44/150; 22.0–36.6)	
	Attock, Bahawalpur, Gujranwala, Kasur, Khanewal, Multan, Okara, Rahim Yar Khan, Vehari	Ticks from sheep and goats	Molecular	Healthy	*A. ovis*, *A. centrale*, *A. marginale*, *A. platys*-like organism, *Anaplasma* sp. BL099-6	38.9 (21/54; 26.2–53.1)	[86]
Khyber Pakhtunkhwa	Charsadda, Mardan, Nowshera, Peshawar	Sheep	Morphological	Suspected of anaplasmosis	*Anaplasma* sp.	29.6 (32/108; 21.0–38.2)	[107]
		Goats				1.7 (1/60; 0.0–8.9)	
	Karak	Sheep	Morphological	Suspected of anaplasmosis	*A. marginale*	22.0 (55/250; 16.9–27.1)	[29]
		Goats				17.2 (43/250; 12.5–21.9)	
		Sheep	Serological *			36.8 (92/250; 30.8–42.8)	
		Goats				32.8 (82/250; 27.0–38.6)	
		Sheep	Molecular			47.2 (118/250; 41.0–53.4)	
		Goats				34.8 (87/250; 28.9–40.7)	
	Peshawar	Sheep	Serological	Healthy	*A. marginale*	24.5 (92/376; 20.1–28.8)	[108]
	Charsadda	Sheep	Serological	Healthy	*Anaplasma* sp.	19.3 (58/300; 14.9–23.8)	[109]
		Goats				25.0 (75/300; 20.1–29.9)	
	District information not provided	Goats	Morphological	Healthy	*A. ovis*	9.6 (7/73; 2.8–16.3)	[110]
	Mardan	Sheep	Morphological	Healthy	*Anaplasma* sp.	13.9 (25/180; 8.8–18.9)	[111]
		Goats				8.3 (15/180; 4.3–12.4)	
		Sheep	Serological			23.9 (43/180; 17.7–30.1)	
		Goats				20.6 (37/180; 14.7–26.5)	
	Bajaur, Khyber, Mohmand, North Waziristan, Orakzai	Ticks from sheep	Molecular	Healthy	*A. centrale*, *A. marginale, A. ovis*	39.1 (9/23; 19.2–59.1)	[22]
		Ticks from goats				35.5 (11/31; 18.6–52.3)	
	Peshawar	Sheep	Morphological and molecular	Suspected of anaplasmosis	*Anaplasma* sp.	28.0 (28/100; 19.2–36.8)	[112]
Sindh	Mirpur Khas	Goats	Morphological	Healthy	*A. marginale*	13.3 (40/300; 9.5–17.2)	[98]

* ELISA: Enzyme-linked immunosorbent assay.

**Table 5:** List of key studies on babesiosis and theileriosis of small ruminants in Pakistan.

State(s)	District(s)	Host(s)/Ticks	Method(s) of Detection	Target Animal Population	Pathogen(s) Detected	% Test-Positive (Proportion; 95% CI)	Reference
Punjab	Lahore	Sheep	Morphological	Suspected of piroplasmosis	*Theileria* sp.	22.0 (44/200; 16.3–27.7)	[27]
			Molecular		*Theileria ovis*	27.5 (55/200; 21.3–33.7)	
					*T. lestoquardi*	7.5 (15/200; 3.8–11.2)	
		Ticks from sheep		Not applicable	*T. ovis*	65.9 (27/41; 51.3-80.4)	
					*T. lestoquardi*	66.7 (30/45; 52.9-80.4)	
	Bahawalnagar, Dera Ghazi Khan, Layyah, Multan, Muzaffargarh	Sheep	Molecular	Healthy	*T. lestoquardi*	8.2 (4/49; 0.5-15.8)	[125]
		Goats				0.0 (0/66; 0.0-5.4)	
	Lahore	Sheep	Morphological	Suspected of piroplasmosis	*Babesia* sp.	23.5 (57/243; 18.1–28.8)	[99]
		Goats				13.5 (51/377; 10.1–17.0)	
	Bahawalnagar, Dera Ghazi Khan, Khanewal, Layyah, Multan, Muzaffargarh, Vehari	Sheep	Molecular	Healthy	*Babesia ovis*	50.0 (20/40; 34.5–65.5)	[118]
		Goats				23.9 (16/67; 13.7–34.1)	
	Lahore	Sheep	Morphological	Suspected of piroplasmosis	*Theileria* sp.	13.9 (38/273; 9.8–18.0)	[123]
		Goats				8.2 (21/256; 4.8–11.6)	
	Sahiwal	Sheep	Morphological	Healthy	*Babesia* sp.	9.7 (30/310; 6.4–13.0)	[100]
	Multan	Sheep and goats	Morphological	Healthy	*Theileria* sp.	3.7 (11/300; 1.5–5.8)	[126]
		Sheep	Molecular		*T. ovis*	15.3 (23/150; 9.6–21.1)	
					*T. lestoquardi*	10.7 (16/150; 5.7–15.6)	
		Goats			*T. ovis*	5.3 (8/150; 2.5–10.6)	
					*T. lestoquardi*	4.0 (6/150; 1.6–8.9)	
	Multan	Sheep and goats	Morphological	Healthy	*Theileria* sp.	12.4 (31/250; 8.3–16.5)	[127]
		Sheep	Molecular			16.0 (25/156; 10.3–21.8)	
		Goats				69.1 (65/94; 59.8–78.5)	
	Attock, Bahawalpur, Gujranwala, Kasur, Khanewal, Multan, Okara, Rahim Yar Khan, Vehari	Ticks from sheep and goats	Molecular	Healthy	*Babesia* and *Theileria* spp.	Not provided	[86]
	Multan	Sheep and goats	Morphological	Healthy	*Theileria* sp.	12.5 (25/200; 7.9–17.1)	[128]
			Molecular			39.5 (79/200; 32.7–46.3)	
	Livestock Experimental Stations, Okara	Sheep	Morphological	Healthy	*B. ovis*	29.0 (58/200; 22.7–35.3)	[28]
					*T. ovis*	37.0 (74/200; 30.3–43.7)	
			Molecular		*B. ovis*	55.0 (110/200; 48.1–61.9)	
					*T. ovis*	7.5 (15/200; 3.8–11.2)	
	Multan	Goats	Morphological	Healthy	*Theileria*	5.4 (25/463; 3.3–7.5)	[54]
			Molecular		*T. ovis,* *T. lestoquardi*	16.0 (74/463; 12.6–19.3)	
	Okara	Sheep	Morphological	Healthy	*Theileria* sp.	16.5 (66/400; 12.9–20.1)	[129]
Punjab and Islamabad Capital Territory	Livestock Experimental Stations located at Attock and Islamabad Capital Territory	Sheep	Morphological	Healthy	*Theileria* sp.	7.4 (7/95; 2.1–12.6)	[57]
		Goats				3.8 (7/184; 1.0–6.6)	
Punjab and Khyber Pakhtunkhwa	Kohat, Multan	Sheep	Molecular	Healthy	*Theileria* sp.	31.7 (26/82; 21.6–41.8)	[88]
		Goats				5.3 (6/114; 1.2–9.4)	
	Multan	Sheep and goats				5.5 (7/128; 1.5–9.4)	
	Kohat					34.7 (25/72; 23.7–45.7)	
	Dera Ghazi Khan, Kohat, Layyah, Multan, Rahim Yar Khan	Sheep	Molecular	Healthy	*T. ovis*	11.1 (11/99; 4.9–17.3)	[130]
		Goats				0.9 (1/111; 0.0–4.9)	
		Sheep and goats	Morphological			1.0 (2/210; 0.1–3.4)	
	Dera Ghazi Khan, Layyah, Multan, Rahim Yar Khan		Molecular			1.7 (2/118; 0.3–6.6)	
	Kohat					10.9 (10/92; 4.5–17.2)	
Khyber Pakhtunkhwa	Charsadda, Mardan, Nowshera, Peshawar	Sheep	Morphological	Suspected of piroplasmosis	*Theileria* sp.	15.7 (17/108; 8.9–22.6)	[107]
					*Babesia* sp.	14.8 (16/108; 8.1–21.5)	
		Goats			*Theileria* sp.	0.0 (0/60; 0.0–7.5)	
					*Babesia* sp.	41.7 (25/60; 29.2–54.1)	
	Lower Dir	Sheep	Molecular	Healthy	*T. annulata,* *T. luwenshuni, T. ovis, Theileria* sp. *MK, Theileria* sp. *OT1*	72.5 (58/80; 62.7–82.3)	[89]
		Goats				40.8 (49/120; 32.0–49.6)	
	Kohat, Peshawar	Sheep	Molecular	Healthy	*T. lestoquardi*	4.5 (2/44; 0.6–15.5)	[131]
		Goats				2.5 (3/121; 0.5–7.0)	
	Khyber, Peshawar	Sheep	Morphological	Healthy	*Babesia* sp.	7.0 (21/300; 4.1–9.9)	[132]
		Goats			*Theileria* sp.	6.0 (18/300; 3.3–8.7)	
	Bannu, Dera Ismail Khan, Tank	Goats	Molecular	Healthy	*Theileria ovis*	9.0 (54/600; 6.7–11.3)	[31]
					*T. lestoquardi*	5.3 (32/600; 3.5–7.1)	
	Bannu, Dera Ismail Khan, Tank	Sheep	Morphological	Healthy	*Theileria* sp.	20 (120/600; 16.8–23.2)	[133]
	Mansehra	Ticks from sheep	Morphological	Not applicable	*B. ovis*	1.5 (3/200; 0.3–4.3)	[48]
		Ticks from goats				1.0 (2/201; 0.1–3.5)	
	Bannu, Dera Ismail Khan, Tank	Sheep	Molecular	Healthy	*T. ovis*	13.0 (78/600; 10.3–15.7)	[30]
					*T. lestoquardi*	9.0 (54/600; 6.7–11.3)	
	Bajaur, Khyber, Mohmand, North Waziristan, Orakzai	Ticks from sheep	Molecular	Healthy	*Theileria* sp.	30.4 (7/23; 11.6–49.2)	[22]
		Ticks from goats				35.5 (11/31; 18.6–52.3)	
		Ticks from sheep			*B. ovis*	0.0 (0/23; 0.0–17.8)	
		Ticks from goats				0.0 (0/31; 0.0–13.7)	
Balochistan	Loralai, Quetta	Sheep	Morphological and molecular	Healthy	*T. ovis*	5.5 (120/2200; 4.5–6.4)	[134]
					*T. lestoquardi*	15.4 (338/2200; 13.9–16.9)	
		Goats			*T. ovis*	6.7 (45/670; 4.8–8.6)	
					*T. lestoquardi*	3.0 (20/670; 1.7–4.3)	

**Table 6:** List of other key tick-borne (including zoonotic) diseases of small ruminants in Pakistan.

State	District(s)	Host(s)	Method(s) of Detection	Target Animal Population	Pathogen(s) Detected	% Test-Positive (Proportion; 95% CI)	Reference
Punjab	Bahawalpur, Bhakkar, Khanewal, Khushab, Layyah, Okara, Rajanpur	Sheep	Serological (ELISA)	Healthy	*Coxiella burnetti* (antibodies)	15.6 (78/500; 12.4–18.8)	[34]
		Goats				15.0 (75/500; 11.9–18.1)	
		Ticks from sheep	Molecular (qPCR)		*Coxiella burnetti* (DNA)	31.0 (9/29; 14.2–47.9)	
		Ticks from goats				7.7 (2/26; −2.6–17.9)	
	Layyah, Muzaffargarh	Sheep	Serological (ELISA)	Healthy	*C. burnetti* (antibodies)	33.2 (90/271; 27.6–38.8)	[33]
		Goats				28.4 (77/271; 23.0–33.8)	
	Attock, Chakwal, DG Khan, Faisalabad, Gujranwala, Lahore, Sahiwal, Sargodha, Sheikhupur	Sheep	Serological (ELISA)	Healthy	*C. burnetti* (antibodies)	17.9% (33/184; 12.4–23.5)	[136]
		Goat				16.4% (46/280; 12.1–20.8)	
Khyber Pakhtunkhwa	Bajaur, Khyber, Mohmand, North Waziristan Orakzai	Ticks from sheep	Molecular (qPCR)	Not applicable	*Rickettsia* (DNA)	73.9 (71/23; 56.0–91.9)	[22]
					*Ehrlichia* (DNA)	8.7 (2/23; 1.1–22.0)	
					*Francisella*-like (DNA)	30.4 (7/23; 11.6–49.2)	
					*Coxiella*-like (DNA)	8.7 (2/23; 1.1–22.0)	
		Ticks from goats			*Rickettsia* (DNA)	83.9 (26/31; 70.9–96.8)	
					*Ehrlichia* (DNA)	3.2 (1/31; 0.0–16.7)	
					*Francisella*-like (DNA)	16.1 (5/31; 3.2–29.1)	
					*Coxiella*-like (DNA)	6.5 (2/31; 1.1–22.8)	
Balochistan	Harnai, Kalat, Killa Abdullah, Khuzdar, Lasbela, Loralai, Pishin, Quetta, Sherani, Sibi, Ziarat, Zhob	Ticks from sheep and goats	Molecular (qPCR)	Not applicable	Crimean-Congo haemorrhagic fever Virus (DNA)	3.8 (20/525; 2.2–5.4)	[61]
	Harnai, Kalat, Killa Abdullah, Khuzdar, Lasbela, Loralai, Pishin, Quetta, Sherani, Sibi, Ziarat, Zhob	Sheep	Serological (ELISA and IFA) and molecular	Healthy	CCHF virus (antibodies)	18.6 (149/800; 15.9–21.3)	[35]
		Goats				4.6 (37/800; 3.2–6.1)	
Sindh	Karachi	Sheep	Serological (CFT)	Not applicable	*C. burnetti* (antibodies)	18.3 (11/60; 8.5–28.1)	[135]
		Goats				4.6 (3/65; 1.0–12.9)	
Punjab, Sindh, Khyber Pakhtunkhwa, and Balochistan	District information not provided	Sheep	Serological (ELISA)	Healthy	CCHF virus (antigen)	32.5 (138/424; 28.1–37.0)	[32]
		Goats				18.9 (83/440; 15.2–22.5)	
Azad Jammu and Kashmir, Balochistan, Gilgit Baltistan, Khyber Pakhtunkhwa, Punjab and Sindh	District information not provided	Ticks from sheep and goats	Next-Generation Sequencing	Not applicable	Several bacterial species (DNA)	Not provided	[20]

ELISA: Enzyme-linked immunosorbent assay; IFA: Indirect fluorescence assay; CFT: Complement fixation test.

## 4. Conclusions and Future Perspectives

To date, most of the previous studies on TTBDis of small ruminants in Pakistan have (i) reported point prevalences of ticks and TBPs, mainly from Punjab and Khyber Pakhtunkhwa provinces, (ii) been conducted in peri-urban areas or near veterinary institutes using convenient and random sampling strategy, and (iii) utilised morphological methods for the identification of TTBPs. Although these studies have provided the information about the prevalence of TTBPs and some seasonal variation of ticks in small ruminants from Pakistan, there are still knowledge gaps about important epidemiological aspects of TTBDis, including (i) the lack of accurate species identification of ticks and TBPs using molecular methods as the microscopic examination has been the most common method of identification which has very low sensitivity and specificity [74,75], (ii) unavailability of data on risk factors, (iii) limited knowledge of TTBPs across different AEZs and production systems, seasonal variation of TBPs, and the efficacy of drugs used against TTBDis and acaricidal resistance.

For TBPs, several studies also used molecular and serological diagnostic methods. However, in most cases, molecular methods were only applied to amplify the target DNA from the positive samples screened by microscopic examination. Moreover, PCR amplicons were only rarely sequenced, and the target pathogens were identified solely based on the visualisation of expected PCR amplicon size on the agarose gel. Additionally, a few studies tested ticks for the presence of TBPs of veterinary (*n* = 6) or zoonotic significance (*n* = 4). Finally, no attempts were made to determine the vector competence of various tick species.

Future research should be directed towards investigating the epidemiology of TTBDis across different AEZs, in different seasons and under various production systems. In developing countries like Pakistan, where resources and laboratory facilities are limited, newly developed field-oriented and low-cost diagnostic methods could be very useful for routine and large-scale surveillance of TTBPs. One such method is LAMP [153]. It is a highly specific, simple, sensitive, robust and rapid method [154] and has the capacity to detect pathogens efficiently in partially processed tick specimens [155,156]. Furthermore, recent innovations of paper-based microfluidics for malaria diagnosis [157] and image-processing based platforms using smartphones for the detection of Chikungunya, Dengue, and Zika viruses [158,159] could be adopted for field diagnosis and surveillance of major TBPs of small ruminants. Concurrently, research should also be focused on unexplored areas of population genomics of TTBPs using high-throughput techniques such as NGS and microfluidics. As discussed earlier, the current focus of research is on the control of TTBDis of large ruminants in this country [72,119,160,161,162], however, in a predominantly mixed-species farming system where small and large ruminants are reared together, sheep and goats could serve as alternative hosts for TTBPs of large ruminants [163,164]. Therefore, for the effective control of TTBDis of livestock in Pakistan, it is imperative to design future studies including common livestock species that are kept in proximity.

Climate change is playing a preeminent role in the expansion of tick population ranges as well as enhancing the pathogen transmission to humans and animals worldwide [165,166,167]. Data-based modelling studies suggest that the changing rainfall patterns and rising environmental temperature would cause a long-term change in the dynamics of TTBPs, resulting in an increased risk of infection in humans and animals [165]. It is likely that developing countries like Pakistan could be exposed to the significant impact of climate change, mainly due to the lack of awareness about mitigation measures, a weak economy and poor institutional capacity to combat this emerging issue [168,169]. In order to design preparatory strategies to tackle climate change in the area of livestock diseases, TTBPs-specific data are required from different mammalian hosts and their habitats from various AEZs of the country.

Studies on assessing the efficacy of various drugs against TTBPs would be pivotal for the identification of potentially effective control methods of TTBDis of livestock in Pakistan. Furthermore, alternative, eco-friendly and sustainable measures (such as multivalent vaccines) should be explored for the control of TTBDis and omics-derived tick microbiome information could be useful for this purpose. Farmer awareness campaigns should also be launched about the rational use of acaricides and risks associated with TTBPs, particularly in case of zoonotic TBPs. Participatory epidemiology (PE) could be very useful for such campaigns as well as for large-scale epidemiological investigations as it has been recently utilised for investigation of bovine health and production constraints in Pakistan [114]. Mobile phone-based applications to process images [170] could also be used for investigations on incidence, prevalence, risk factors and control practices for TTBDis of small ruminants as well as assessing related knowledge within communities in Pakistan and other parts of the world.

Overall, this review has demonstrated that the prevalence of TTBDis in Pakistani small ruminants is high and emphasises the need for further intensive research on the epidemiology, ecology, population genomics and control of TTBPs. The use of advanced but ‘practical’ diagnostic tools will be critical in attaining an improved understanding of interactions among vectors, microbiomes, mammalian hosts and the environment, and should guide the development of integrated and sustainable control of TTBDis through the One Health perspective. It is believed that such a strategy would provide effective control of TTBDis of small ruminants and benefit the resource-poor farmers in Pakistan and elsewhere to address the challenge of food security.
